# Menstrual Cycle Phase Modulates Emotional Conflict Processing in Women with and without Premenstrual Syndrome (PMS) – A Pilot Study

**DOI:** 10.1371/journal.pone.0059780

**Published:** 2013-04-24

**Authors:** Jana Hoyer, Inga Burmann, Marie-Luise Kieseler, Florian Vollrath, Lydia Hellrung, Katrin Arelin, Elisabeth Roggenhofer, Arno Villringer, Julia Sacher

**Affiliations:** 1 Department of Cognitive Neurology, Max Planck Institute for Human Cognitive and Brain Sciences, Leipzig, Germany; 2 Clinic for Cognitive Neurology, University Hospital Leipzig, University of Leipzig, Leipzig, Germany; Max Planck Institute of Psychiatry, Germany

## Abstract

**Background:**

Premenstrual syndrome (PMS) is characterized by a cluster of psychological and somatic symptoms during the late luteal phase of the menstrual cycle that disappear after the onset of menses. Behavioral differences in emotional and cognitive processing have been reported in women with PMS, and it is of particular interest whether PMS affects the parallel execution of emotional and cognitive processing. Related to this is the question of how the performance of women with PMS relates to stress levels compared to women without PMS. Cortisol has been shown to affect emotional processing in general and it has also been shown that women with severe PMS have a particular cortisol profile.

**Methods:**

We measured performance in an emotional conflict task and stress levels in women with PMS (n = 15) and women without PMS (n = 15) throughout their menstrual cycle.

**Results:**

We found a significant increase (p = 0.001) in the mean reaction time for resolving emotional conflict from the follicular to the luteal cycle phase in all subjects. Only women with PMS demonstrated an increase in physiological and subjective stress measures during the luteal menstrual cycle phase.

**Conclusions:**

Our findings suggest that the menstrual cycle modulates the integration of emotional and cognitive processing in all women. Preliminary data are supportive of the secondary hypothesis that stress levels are mediated by the menstrual cycle phase only in women with PMS. The presented evidence for menstrual cycle-specific differences in integrating emotional and cognitive information highlights the importance of controlling for menstrual cycle phase in studies that aim to elucidate the interplay of emotion and cognition.

## Introduction

Up to 75 percent of women experience some degree of premenstrual syndrome (PMS) during their reproductive years [1], [2]. This condition includes somatic symptoms, such as fatigue, appetite-changes, and low energy, and affective symptoms, such as irritability, depressed mood, anxiety, and impulsive behavior [2], [3]. Typically, symptoms remit within a few days after the onset of menstruation. Approximately 10 percent of women with PMS experience a very severe form called premenstrual dysphoric disorder (PMDD), with similar prevalence in the United States [4], Canada [5], [6], Europe [7], India [8], Nigeria [9], and Japan [10]. As recently stated by Epperson and colleagues [11], PMDD shows comparable rates in Caucasians and African Americans in the United States [12], and symptoms appear to be relatively stable over time [7], [13]. PMDD is included in the current Diagnostic and Statistical Manual of Mental Disorders, Fourth Edition (DSM-IV), Text Revision; the depressed mood that women with PMDD experience corresponds in severity to a major depressive episode (MDE) [14]. This emphasizes the interrelatedness of PMS/PMDD and depression, an association which is further supported by evidence revealing higher comorbidity and lifetime prevalence for major depressive disorder (MDD) in women experiencing PMS and PMDD [15], [16]. Transitions between PMS and PMDD are fluid and a common cause for both entities has been assumed [17].

However, the etiology of PMS and PMDD is largely unknown. Because PMS symptoms are closely related to the menstrual cycle and only affect women of reproductive age, sex hormones have been suggested to play a causative role. However, a large body of evidence indicates that women who are vulnerable to premenstrual mood changes do not have abnormal levels of sex hormones [18], [19]. Thus, it appears that women with PMS and PMDD show an abnormal response to normal sex hormone changes across the menstrual cycle [20]. As reviewed by Epperson et al. [11], genetic and psychosocial risk factors, such as a preexisting major mood disorder, history of sexual abuse, exposure to domestic violence, and a stressful work, home, or school environment have been implicated.

An interesting line of work has implicated the stress hormone cortisol due to the observation of an altered timing of cortisol profiles in women with PMDD compared to women without PMDD during the follicular menstrual cycle phases [21]. Dysregulation of the hypothalamic-pituitary-adrenal (HPA) axis has been demonstrated in major depressive disorder and has been speculated to have an important role in the induction of sadness and impaired mood regulation [22], [23]. In a non-clinical population, cortisol levels have been shown to correlate with depressed mood and with poorer performance in an emotional processing task [24]. As endocrinological measures, such as salivary cortisol levels, thus seem useful to include in the research of premenstrual affective disorders, they would be more promising when combined with behavioral measures in order to better grasp the complexity of the potentially abnormal response to sex hormone changes that has been postulated to occur in women with PMS.

Investigators agree that premenstrual affective disorders, such as PMS, provide an unprecedented opportunity to study how changes in sex hormones impact the processing of emotional information and mood regulation on a behavioral level (for a detailed review see: [20]). Several lines of evidence support this concept: a subtle impairment in the identification of affective facial expressions has been observed for women with PMDD in the luteal versus the follicular cycle phase [25], as well as higher negative affect [26] and higher physiological reactivity [27]. Also, in the luteal phase, both women with PMDD and women without PMDD have demonstrated lower performance in a task that requires focused attention and high vigilance [26]. Women with clinical level premenstrual affect-disturbances showed enhanced bias to negative information, decreased bias to positive information, and diminished inhibitory control [28]. This was assessed in an emotional linguistic Go-No Go task designed to assess the interaction between emotion and motor inhibition using negative, neutral, and positive words as stimulus material. This task requires the subject to press a button when a word in normal font (go trial) appears and to withhold a response when a word in italicized font (no-go trial) appears.

Additional preliminary evidence for changes in behavioral patterns across the menstrual cycle – including changes in selective attention, cognitive flexibility and processing speed – stems from a study that applied the Trail Taking Test and the Stroop task in women with PMS and a control group of women without PMS [29]. In the Trail Making Test, the assignment is to draw a consecutive line connecting alternating numbers and letters in sequence (1-A-2-B-3-C…). Performance in this task was better during the follicular phase of the menstrual cycle for all subjects, with an overall better performance of controls versus women with PMS. The Stroop task assesses performance to correctly name a color during interference created when the name of a color (e.g., “green”) is printed in a color not denoted by the name (e.g., the word “green” printed in blue ink instead of green ink). In both groups, more errors were made during the late luteal phase, but color naming was faster during this period, suggesting a degree of disinhibition and impulsivity in all women during the late luteal phase.

While these findings for emotional and cognitive processing represent important contributions to our understanding of different aspects of behavior that might be influenced by the menstrual cycle, it is the parallel execution of both emotional and cognitive processing that is required for optimal performance in a situation of emotional conflict. However, little is known about whether emotional conflict processing is affected by the menstrual cycle phase and how performance in women with PMS differs from that in women without PMS. Using an elegant modification of the traditional Stroop task [30], involving the identification of an emotional facial expression without being distracted by a word expressing an emotion written across that face instead of the indentification of a color, allows for the assessment of emotional incongruence. While this paradigm has been successfully applied to studying emotional conflict in depression [31], anxiety [31], and panic disorder [32], as well as menopausal transition [33], it has never been used in a population with premenstrual affective disorder.

In the present study, we investigated whether performance in an emotional conflict task and stress levels are altered by the menstrual cycle in a group of women affected by PMS (hereafter referred to as the PMS group) compared to a control group not affected by PMS (hereafter referred to as the control group). Based on the research outlined above, our main hypothesis is that performance of an emotional conflict task will be impaired in the PMS group reflected by an increase in reaction time during the emotional interference condition in women with PMS compared to controls. We further hypothesize that we will not detect any significant impact of menstrual cycle phase on the parallel execution of emotional and cognitive processing in the control group. Thus, in the control group we hypothesize reaction time during emotional interference not to reveal any significant differences between the late luteal and the late follicular cycle phase. As a secondary and more exploratory aim, we expect stress measures, both self-reported stress scores and levels of salivary cortisol, to increase in the PMS group in the late luteal cycle phase compared to the late follicular cycle phase, while we expect no such changes to be observed in stress measures in the control group.

## Methods

### 1. Subject selection

We investigated 30 females (mean age  = 26±4 years; range  = 20–35 years). Subjects were healthy, medication-free, reported regular menstrual cycles, did not use hormonal contraception, were without any current or previous history of psychiatric illnesses (the Structured Clinical Interview for DSM-IV [34] was used to rule out any Axis I disorders), had no history of gynecological pathology, were ≥1 year post-partum or never pregnant, and not currently breast feeding. Subjects were recruited through advertisements and flyers in local universities, libraries, the local university clinic, and physicians' offices. Participants were screened over the telephone and scheduled for an on-site visit at the Max Planck Institute of Cognitive and Brain Sciences, Department of Neurology in collaboration with the Day Clinic of Cognitive Neurology, University Clinic Leipzig, to determine study eligibility, using medical history and physical examination, which included a brief neurological examination. To monitor the accuracy of individual reporting, the information subjects provided was checked for any inconsistencies regarding length of menstruation, abnormal mid-cycle bleeding and length of cycle by an independent research administrator for at least 3 months prior to testing and were contacted to confirm the timing of menses-onset following testing. In total, 59 subjects were scheduled for an on-site visit, 49 subjects met eligibility criteria and 30 subjects completed the entire protocol. The reasons for discontinuation of the study for the 19 subjects (12 controls, 7 women with PMS) who initially met eligibility but did not complete the entire protocol were the following: inconsistencies/lack of compliance with menstrual cycle reports or scheduling of assessments (12), decision to start oral contraceptives (4), positive pregnancy test (2), and an accident resulting in leg-fracture (1). Before entry into the study, prospective participants were screened using the German version of the premenstrual symptoms screening tool (PSST) [5], [35], which is a valid and reliable instrument for PMS/PMDD screening, and assigned to either the PMS group (*n* = 15) or the control group (*n* = 15). Demographic details of the subjects can be found in Table 1; groups were matched for degree of education, profession and parity. All participants gave written consent to participate. Study and recruitment procedures were carried out in accordance with the Declaration of Helsinki and approved by the research ethics board of the University of Leipzig. All women were tested at the late follicular and the late luteal phase of the menstrual cycle; detailed information on day tested and menstrual cycle length is provided in Table 1. The order of the menstrual cycle phase during which the emotional Stroop task was administered was counter-balanced. At each time of testing, saliva samples for the determination of sex hormones (estradiol, progesterone, testosterone) and cortisol were collected. To guarantee a clean saliva sample, participants had to refrain from caffeine, eating, drinking, and brushing their teeth for two hours before the sample was taken. Cortisol and the sex hormones were determined with a competitive luminescence immunoassay (CLIA) by IBL (Hamburg, Germany). The determinable range in saliva was as follows: for cortisol 0.005–4 µg/dL, for progesterone 2.6–1000 pg/mL, for testosterone 1.8–500 pg/mL, and for 17β-estradiol 0.3–64 pg/mL. Intra-assay coefficients of variation (CV) were 9% for 17β-estradiol, 5% for progesterone, 2% for testosterone and 5% for cortisol. Inter-assay CVs were 15% for 17β-estradiol, 8% for progesterone, 7% for testosterone and 4% for cortisol. At each time of testing the Beck Depression Inventory (BDI) [36], the Hamilton Depression Scale (HAM-D) [37], and the Perceived Stress Scale (PSS) [38] were administered. Four subjects in the PMS group and two subjects in the control group did not return the PSS questionnaire at one point of testing and were excluded from the subjective stress score analysis.

**Table 1:** Sociodemographic characteristics (mean ± SD) of both groups.

	*PMS*	*Controls*
*N*	15	15
Age (years)	26±4	27±4
BMI (kg/m^2^)	23±4	24±4
Examined day of menstrual cycle for follicular phase	13±2	12±2
Examined day of menstrual cycle for luteal phase	27±3	27±2
Cycle length (days)	29±3	28±2

*Note.* BMI  =  Body Mass Index; PMS  =  Premenstrual Syndrome.

No significant differences in age, BMI, menstrual cycle length or days of menstrual cycle tested.

### 2. The Emotional Stroop Task (EST)

We employed a German version of the emotional conflict paradigm, as described by Etkin et al. [30], which has been used previously in a German population [32]. In this paradigm, combinations of an emotional face in the background (happy or fearful expression, from the Ekman faces set [39]) and the word “GLÜCK” or “ANGST” (German for “HAPPINESS” and “FEAR”, respectively) printed across the face in bold, red capital letters are presented. Trials were displayed for 4000 msec with a jittering interstimulus interval (4.00±0.4 sec, range 3–5 sec; Presentations software, Neurobehavioral Systems, Albany, USA), thereby introducing a randomized variability in stimulus presentation that has been associated with increased vigilance to such a task. One run consisted of 190 trials in sections of 22 blocks and 20 breaks of 4 seconds. In between face presentations, a fixation cross (a cross hair displayed on the screen for orientation) was shown. Depending on the congruence between face expression and word, trials were classified as congruent (C) or incongruent (I). Order types were counterbalanced across the experiment. To avoid priming effects, direct repetitions of the same face and repetitions of the same face-word-distractor combination (e.g., happy face, word “fear”) were excluded, as has been done previously [31], [32]. Participants were instructed to identify the face expression and answer as quickly and precisely as possible by pressing the right (happy face) or left (fearful face) answer button with their index finger.

### 3. Data Analysis

Reaction times collected during the emotional Stroop experiment were analyzed. Error trials (wrong answers, omissions and double responses) and trials with outlier reaction times (> three interquartile lengths below the 1^st^ quartile or > three interquartile lengths above the 3^rd^ quartile) were excluded from any reaction time calculations. For the accuracy calculations, all types of errors were considered. Statistical analysis was performed using the IBM SPSS Statistics 19 program (SPSS Inc, Chicago, IL). Normal distribution was tested with a One-Sample-Kolmogorov-Smirnoff test. We computed independent *t*-tests for comparisons of menstrual cycle phases (follicular versus luteal) between groups, and paired-*t* tests for within-group comparisons. In a second step, we analyzed interactions, applying a general linear model (GLM) with repeated measures. The within-subject factor was the reaction time according to cycle phase: follicular or luteal. The between-subjects factor was the group differentiation in the PMS and control groups. The applied contrast was the Helmert contrast. Correlations between reaction times and cortisol levels were calculated with Spearman rank order correlation coefficients. For cortisol, logarithmic values (natural log) were computed before analyses to normalize the cortisol distribution [40].

## Results

There were no significant differences in the average day of testing in the follicular cycle phase between the PMS group (day  = 13, *SD*  = 2) and the control group (day  = 12, *SD*  = 2), or in the late luteal cycle phase (PMS group: day  = 27, *SD*  = 3; control group: day  = 27, SD  = 2; details given in Table 1), or the average time of day when testing occurred (PMS group: Central European Time (CET) mean ± *SD*  = 12.50±114 min; control group: CET mean ± *SD*  = 12.06±102 min). Progesterone levels showed a significant rise from the follicular and luteal phase within subjects (PMS group, *p* = 0.03; control group, *p* = 0.02). We did not find any significant differences for any salivary sex hormone levels (estradiol, progesterone, and testosterone) between the PMS group and the control group; details are given in Table 2.

**Table 2:** Salivary sex hormone levels (mean ± SD) in follicular and luteal cycle phases for both groups.

*Hormone*	*Cycle Phase*	*PMS*	*Controls*
Estradiol	follicular	7±8	5±2
(pg/mL)	luteal	9±10	7±5
Progesterone	follicular	53±24	61±65
(pg/mL)	luteal	106±96	148±139
Testosterone	follicular	11±9	21±21
(pg/mL)	luteal	16±11	13±8

*Note.* PMS  =  Premenstrual Syndrome.

### 1. Psychopathological assessment

**Figure 1:**
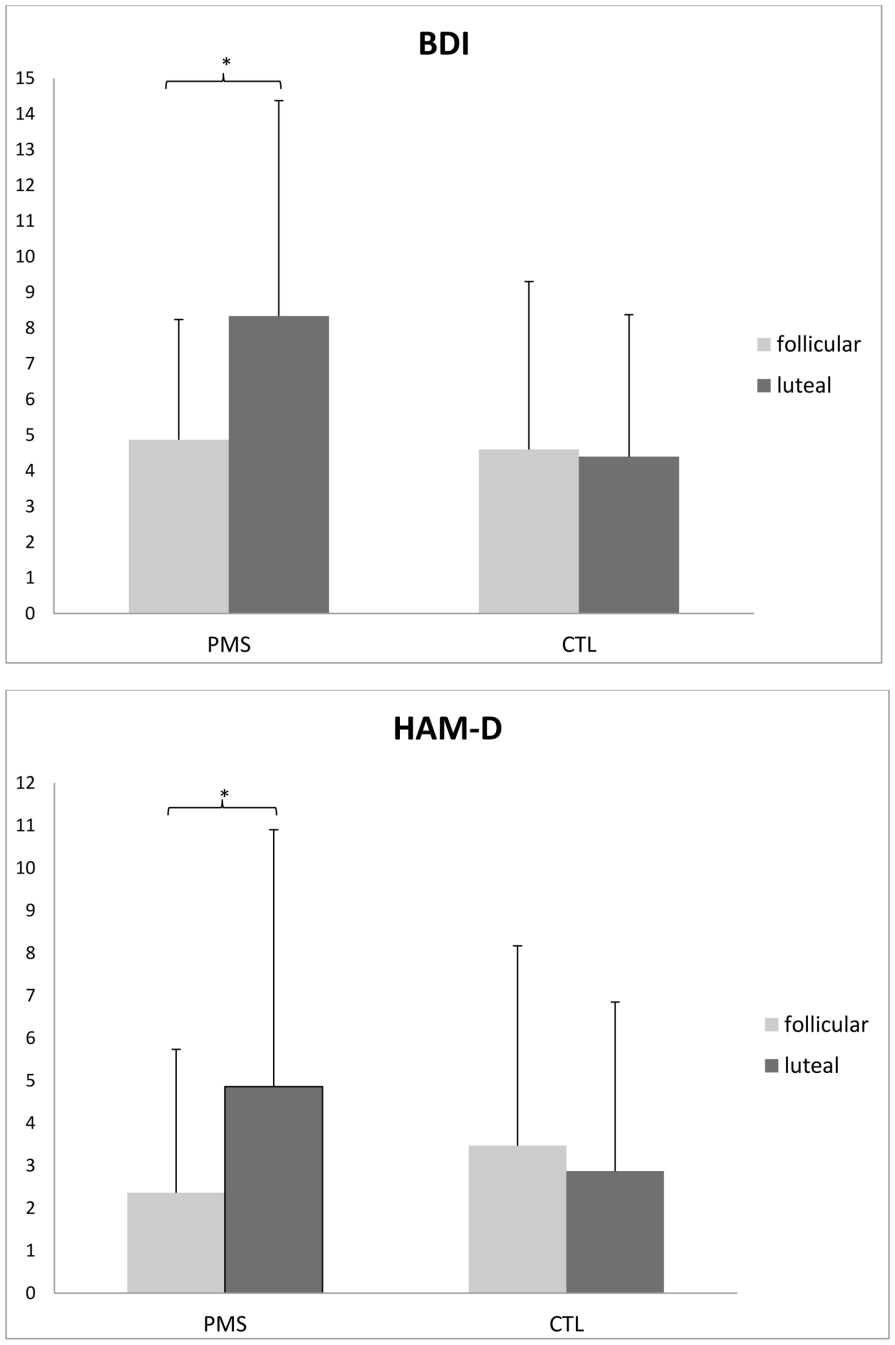
Affective symptoms in PMS subjects and control subjects across the menstrual cycle. **Top Panel:** Beck Depression Inventory (BDI) scores in women with premenstrual syndrome (PMS) are significantly increased (*: *p* = 0.025) in the luteal cycle phase (dark grey) compared to the follicular phase (light grey) but not in women without PMS (CTL). Bars represent one standard deviation. **Bottom Panel:** Hamilton Depression Scale (HAM-D) scores are significantly increased in women with PMS (*: *p* = 0.001) in the luteal cycle phase (dark grey) compared to the follicular phase (light grey) but not in women without PMS (CTL). Bars represent one standard deviation.

Mood ratings in the PMS group and the control group are shown in Figure 1. As expected, the PMS group showed a luteal phase increase in the BDI scale (*t*_14_ = −2.51, *p* = 0.025) and low or absent symptoms in the follicular phase. The control group showed low or absent symptoms in both phases (*t*_14_ = 0.25, *p* = 0.81). Differences in the BDI scale between the PMS and control groups were significant in the luteal phase (*t*_28_ = −2.11, *p* = 0.04) but not in the follicular phase (*t*_28_ = 0.18, *p* = 0.86). HAM-D scores were significantly increased in the PMS group in the luteal cycle phase compared to the follicular phase (*t*_14_ = −4.6, *p* = 0.001) and symptoms in the follicular phase were low or absent. The control group showed low or absent symptoms in both phases (*t*_14_ = 1.15, *p* = 0.27). Differences in the HAM-D scale between the PMS and control groups were significant in the luteal phase (*t*_28_ = 2.21, *p* = 0.035) but not in the follicular phase (*t*_28_ = −1.06, *p* = 0.3).

### 2. Emotional Conflict Task

The main outcome variables for task performance were reaction time and accuracy in determining the facial expression during congruent and incongruent conditions. Results of the task reaction time in the PMS group and the control group were as follows: the PMS group resolved the incongruent condition faster than the control group in the follicular cycle phase (*t*_28_ = −2.34, *p* = 0.03) but not in the luteal cycle phase (*t*_28_ = −1.35, *p* = 0.19). We found a tendency for a menstrual cycle effect, with slower reaction times in the luteal phase of the PMS group (*t*_14_ = −1.99, *p* = 0.07) but not the control group (*t*_14_ = −1.27, *p* = 0.23). When we tested the group by cycle interaction in a general linear model, we observed a trend for the group main effect: the PMS group resolved the incongruent condition faster than the control group (*F*_1,28_ = 3.51, *p* = 0.08). Similarly, a shorter reaction time was evident in the congruent condition for the PMS group compared to the control group in the follicular phase (*t*_28_ = −2.06, *p* = 0.05) but not in the luteal phase (*t*_28_ = −1.18, *p* = 0.25). During congruent trials, we did not observe a menstrual cycle effect in the PMS group (*t*_14_ = −0.79, *p* = 0.44) or the control group (*t*_14_ = 0.01, *p* = 0.99). In the group by menstrual cycle interaction (GLM), there was a trend for the group main effect, indicating that the PMS group tended to resolve congruent trials faster than the control group (*F*_1,28_ = 2.82, *p* = 0.10).

**Figure 2:**
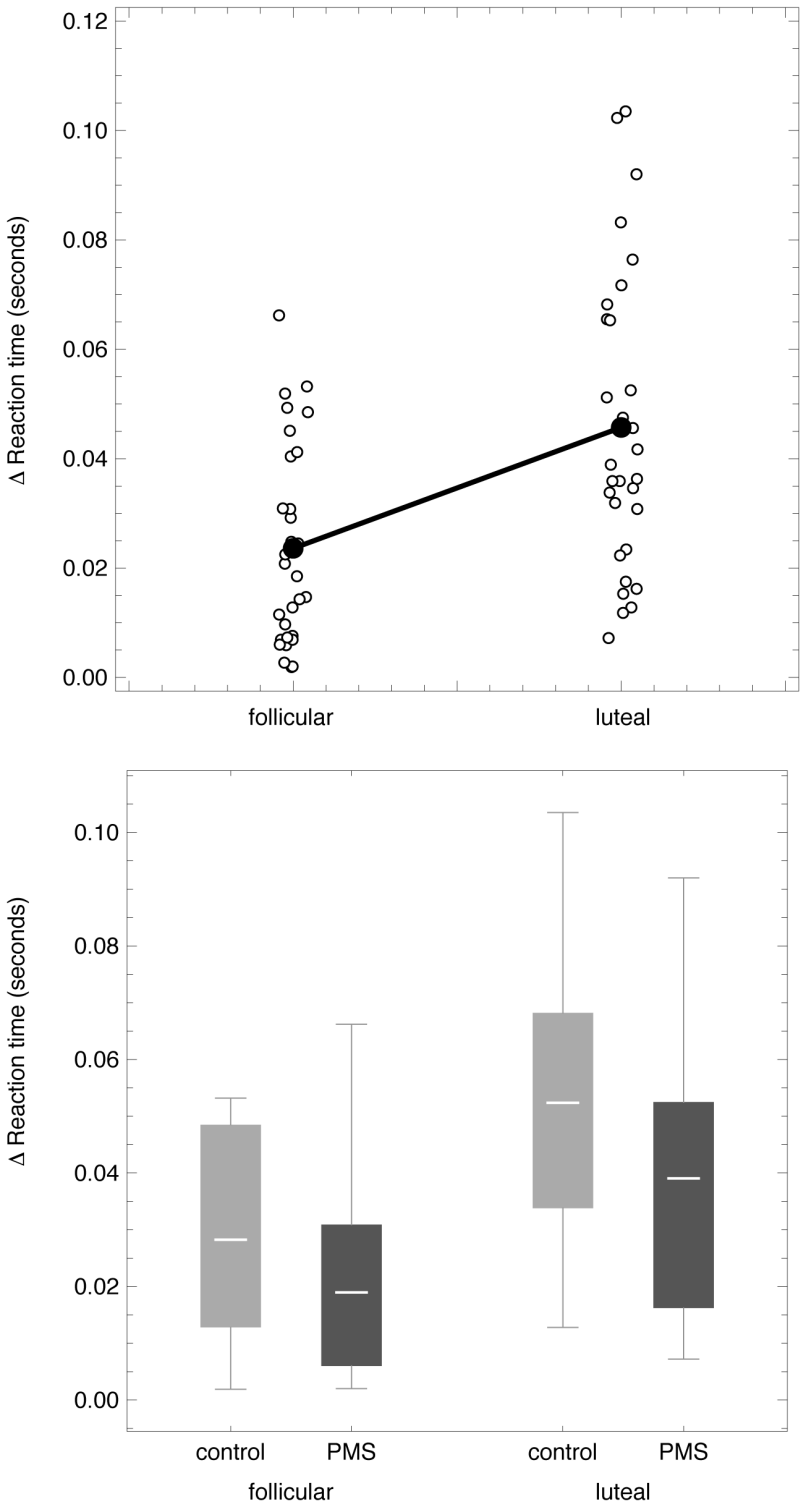
Resolution of Emotion Conflict differs according to menstrual cycle phase. **Top panel:** Scatter plots show individual differences in reaction time (sec) between the incongruent and congruent condition of the emotional Stroop task in the follicular and the luteal cycle phase in all subjects (mean reaction time shown in black). These data indicate a significant increase (*p* = 0.001) in mean reaction time for resolving emotional conflict between the follicular and the luteal cycle phase in all subjects. **Bottom panel:** Boxplots-bars show mean differences in reaction time (sec) between the incongruent and congruent condition of the emotional Stroop task in the follicular and the luteal cycle phase split by subject group (dark grey  =  PMS group, light grey  =  control group). Whiskers represent minimum and maximum of data-range. On average, the PMS group tended to show a faster mean reaction time compared to the control group (follicular phase: PMS group mean ± SD  = 0.02±0.02 sec, Control group mean ± SD  = 0.03±0.02 sec; luteal phase: PMS group mean ± SD  = 0.04±0.03 sec, Control group mean ± SD  = 0.05±0.03 sec), however, overlap exists and this difference is not significant (follicular phase: *p* = 0.17; luteal phase: *p* = 0.19).

To assess the emotional face-word interference effect across the menstrual cycle, we then computed the mean differences in reaction time between the incongruent and the congruent conditions, as shown in Figure 2. Our data reveal a significant increase (*t*_29_ = −3.9, *p* = 0.001) in the mean reaction time for resolving emotional conflict from the follicular to the luteal cycle phase in all subjects (Figure 2, top panel). On average, the PMS group tended to show a faster mean reaction time compared to the control group (follicular phase: PMS group mean  = 0.02±0.02 sec versus control group mean  = 0.03±0.02 sec; luteal phase: PMS group mean  = 0.04±0.03 sec versus control group mean  = 0.05±0.03 sec), however overlap exists (Figure 2, bottom panel) and this difference is not significant (follicular phase: *t*_28_ = 1.4, *p* = 0.17; luteal phase: *t*_28_ = 1.36, *p* = 0.19). As observed previously, accuracy rates were high for both conditions (above 89 percent in both groups for each menstrual cycle phase) and did not reveal any significant differences.

### 3. Measures of acute physiological and subjective stress reactivity

**Figure 3:**
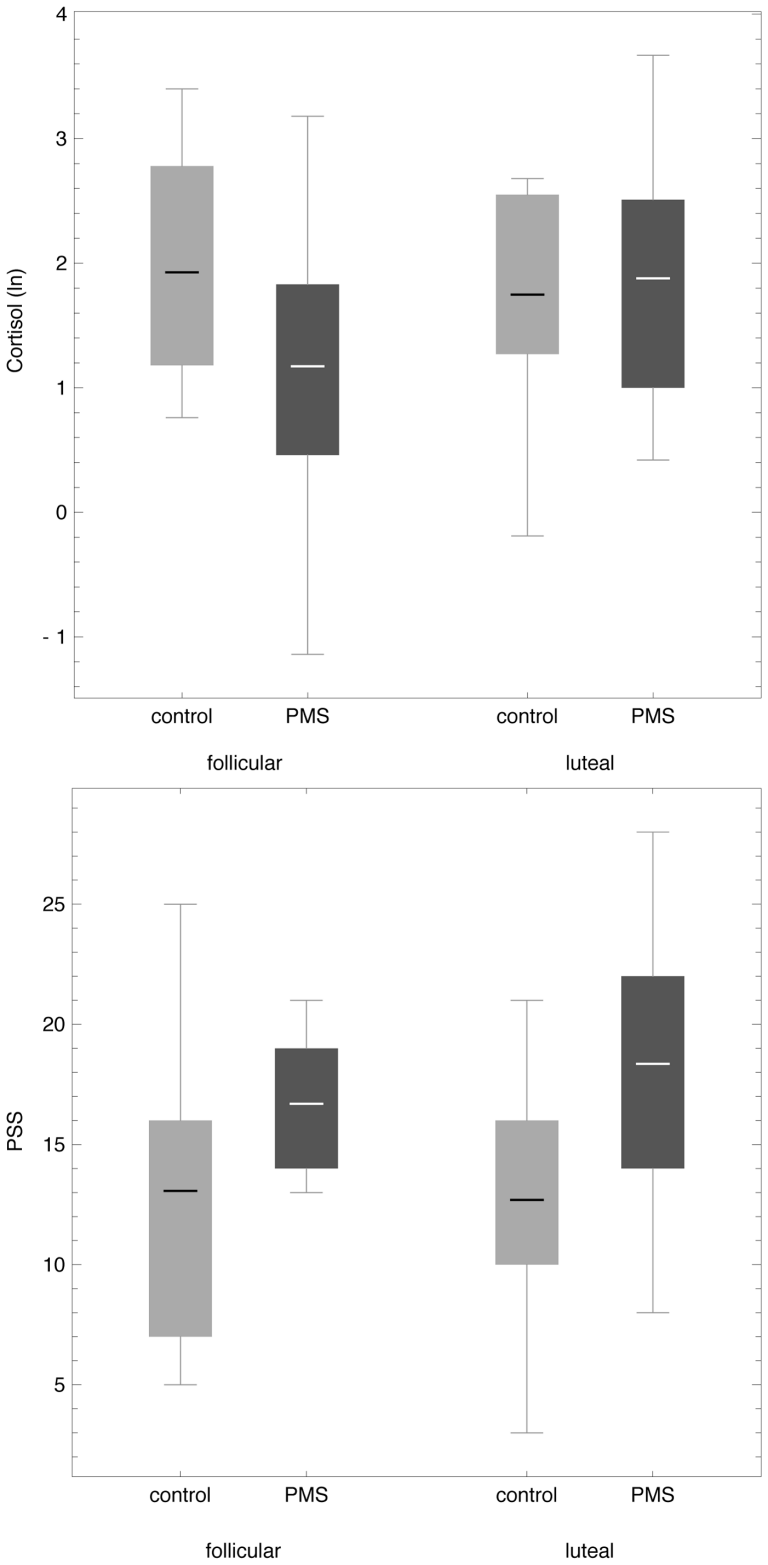
Heightened physiological and subjective stress levels in women with premenstrual syndrome (PMS) in the luteal menstrual phase. **Top Panel:** Boxplots-bars show mean salivary cortisol values (ln) comparing the follicular and the luteal cycle phase in PMS subjects (dark grey) versus control subjects (light grey). Whiskers represent minimum and maximum of data-range. On average, salivary cortisol levels increased from the follicular to the luteal cycle phase in the PMS group (*p* = 0.04). **Bottom panel:** In the Perceived Stress Scale (PSS), the PMS group (dark grey) displayed a similar trend for an increase from the follicular to the luteal cycle phase (*p* = 0.20) as depicted by boxplot-bars. On average, the PMS group (dark grey) tended to report higher subjective acute stress reactivity compared to the control group (light grey), revealing a significant difference for the luteal cycle phase (*p* = 0.01). Whiskers represent minimum and maximum of data-range.

We found an increase in salivary cortisol levels from the follicular to the luteal cycle phase in the PMS group (*t*_14_ = −2.29, *p* = 0.04) (top panel, Figure 3). In the subjective stress reactivity measure, namely the Perceived Stress Scale, we detected a trend for a similar increase from the follicular to the luteal cycle phase in the PMS group (*t*_11_ = −1.36, *p* = 0.20). The PMS group tended to report higher subjective acute stress levels compared to the control group, revealing a significant difference for the luteal cycle phase (*t*_23_ = −2.78, *p* = 0.01) (bottom panel, Figure 3). There were no significant correlations between salivary cortisol levels and emotional Stroop task performance across the whole sample.

## Discussion

The primary goal of this study was to investigate a potential effect of menstrual cycle phases on parallel execution of cognitive and emotional processing in women affected by PMS compared to women not affected by PMS. We were particularly interested in whether women with PMS would demonstrate an increase in reaction time in the late luteal phase of the menstrual cycle, the time when they are symptomatic. Our data support the tendency of women with PMS to resolve an emotional conflict paradigm more slowly shortly before menses sets in, thus confirming our main hypothesis. As expected, subjects with PMS reported significantly increased scores in depression ratings during the luteal cycle phase while subjects without PMS showed low or absent symptoms in both phases (Figure 1). Heightened depression scores in the PMS group were consistent with PSST ratings, thereby confirming a symptom phase of irritability, anxiety, despair, and depression during the late luteal menstrual cycle phase. While it was also expected that, during the symptomatic luteal cycle phase, subjects with PMS would show a trend for a weaker performance when resolving the emotional interference paradigm than during the late follicular cycle phase when they are not symptomatic, it is interesting that we observed overall faster reaction times in subjects with PMS compared to subjects without PMS (Figure 2). Acknowledging that the effect was only moderate and limited by sample size and inter-subject variability, it is nevertheless an interesting observation, particularly when viewing subclinical premenstrual mood changes as an indicator for a system in distress that is still able to compensate in order to avoid any significant behavioral impairment when solving a task that requires parallel processing of emotional and cognitive stimuli.

We further hypothesized reaction time during emotional interference not to reveal any significant differences between the late luteal and the late follicular cycle phase in the control group. However, performance data for control subjects were not consistent with this hypothesis: we observed a significant increase in mean reaction time for resolving emotional conflict from the follicular to the luteal cycle phase in all subjects including the control group (Figure 2). These results suggest that the menstrual cycle is capable of modulating emotional interference in healthy premenopausal women regardless of whether premenstrual mood changes occur. The subtle fluctuations of sex hormones throughout the female menstrual cycle have previously been demonstrated to account for differences in the perception of emotionally salient stimuli, as reviewed by [41]. Our results are in line with these and other findings regarding the menstrual cycle phase: a trend for better performance in the recognition of facial expression has been observed during the follicular and ovulation phases compared to the luteal menstrual cycle phase [42]–[44]. While these differences in emotional recognition are important to note, it is the parallel execution of emotional and cognitive processing that is required for successful social interaction. PMS interferes in situations of social interaction [45]. Our data from an emotional interference task, which has been demonstrated to be a useful paradigm in the investigation of parallel execution of emotional and cognitive processing [30], [32], extend reports that emotion recognition is affected by menstrual cycle phase: our results reveal that women are more successful in the parallel processing of emotional and cognitive information in the follicular cycle phase compared to the luteal cycle phase (Figure 2).

As a secondary and exploratory line of observation, we obtained preliminary measures of subjective and objective stress to explore whether women with PMS would display any substantial intra-individual changes in stress patterns across the menstrual cycle. While menstrual cycle phase differences in the performance of emotional processing tasks have been linked to salivary cortisol variations over the menstrual cycle in women without PMS [46] and heightened levels of cortisol have been reported for women with PMS [47], [48], several other studies report an absence of typical cortisol patterns for women with PMS [49]–[51] or a decrease in cortisol levels compared to healthy controls [52], [53]. One reason for the difficulty in bridging all these seemingly inconsistent findings can be found in the methodology: the menstrual cycle phases that were compared and the method used to determine menstrual cycle phase differed across studies, as did the time of day for sample collection (cortisol secretion is lower in the evening), the sample medium (CSF, urine, plasma, saliva), the sample size (*n* = 2–42), the groups and characterization (severity of symptoms differed, some studies did not include a non-PMS control group, and the assessment tools used to identify premenstrual symptoms differed). While these are all aspects that need to be considered when reviewing the existing literature, Odber et al. [53] have proposed a compelling concept to consolidate the seemingly conflicting findings: that an increase in baseline cortisol levels in a subclinical sample could represent a physiological and healthy response to the stressful situation of PMS symptoms (albeit mild to moderate), a compensation mechanism protecting a healthy system from further imbalance. This would explain why the increases in cortisol that have been found in groups with a subclinical level of premenstrual mood changes could not be extended to PMDD: a clinical level disorder associated with a pronounced and more sustained imbalance of the HPA axis [21], [52]. Our exploratory observation of an increase in a single sample of salivary cortisol levels in the late luteal phase compared to the follicular phase in a pilot sample of women with PMS is consistent with this theory and in line with the marked increase in subjective stress that PMS subjects reported for this phase (Figure 3).

Several caveats are important to acknowledge in the interpretation of our results. First, the levels of significance obtained are in the modest range, which is likely explained by the limited sample sizes. While the menstrual cycle effect on reaction time during successful emotional conflict resolution is robust, the signals described for between-group effects during the emotional conflict paradigm, cortisol and subjective stress levels do not survive statistical correction for multiple comparisons and therefore warrant further investigation in a larger sample. Second, the determination of menstrual cycle phase was based on the individual diaries of subjects. To monitor the accuracy of individual reporting, subjects had to report menstrual cycle diaries for length of menstruation, abnormal mid-cycle bleeding and length of cycle for at least 3 months prior to testing, were checked for any inconsistencies by an independent research administrator and were contacted to confirm the timing of menses-onset following testing. Collected salivary samples for estradiol and progesterone levels were in the expected ranges for the designated menstrual cycle phases. Although additional validation of these self-reports stems was obtained from the observed rise of progesterone within the expected range, with a clear distinction between follicular and luteal phase (Table 2), it would have been preferable to also perform an ovary ultrasound to confirm ovulation and menstrual cycle phase or to assess lutropin peaks in urine for precise determination of ovulation. Finally, cortisol levels represent only one aspect of HPA axis function and inter-individual cortisol differences are considerable [54]. To obtain a valid assessment of baseline cortisol levels across the menstrual cycle, Nepomnaschy et al. suggest collecting 10–14 samples at multiple times in a longitudinal design [55]. We acknowledge this by limiting our interpretation to the within-group change of cortisol in the PMS group and conclude that this preliminary finding is of exploratory nature and needs further investigation in a larger sample following an assessment protocol as suggested by Nepomnaschy and colleagues [55].

In conclusion, our findings indicate a considerable modulating effect of the menstrual cycle on the parallel execution of emotional and cognitive processing in healthy women reporting regular menstrual cycles. The significantly faster resolution of an emotional conflict task during the follicular menstrual cycle phase compared to the late luteal menstrual cycle phase points towards a mediation of the integration of emotional and cognitive information by subtle fluctuation of sex hormones, possibly influenced by the HPA axis. The present findings emphasize the importance of considering menstrual cycle phase in the design of studies investigating the interplay of cognition and emotion. Furthermore, our data support the concept that subclinical PMS is a potential indicator of distress in a system that is still capable of compensating for the subjective stress associated with monthly mood changes. This study suggests that women with subclinical PMS represent a population of particular interest to studies that endeavour to reduce lifetime prevalence rates of depression in women.
